# Evaluation of the EpiCore outbreak verification system

**DOI:** 10.2471/BLT.17.207225

**Published:** 2018-03-16

**Authors:** Taryn Silver Lorthe, Marjorie P Pollack, Britta Lassmann, John S Brownstein, Emily Cohn, Nomita Divi, Dionisio Jose Herrera-Guibert, Jennifer Olsen, Mark S Smolinski, Lawrence C Madoff

**Affiliations:** a International Society for Infectious Diseases, 9 Babcock Street, Brookline, Massachusetts, 02446, United States of America (USA).; b HealthMap, Harvard Medical School, Boston’s Children’s Hospital, Boston, USA.; c Ending Pandemics, San Francisco, USA.; d Training Programs in Epidemiology and Public Health Interventions Network, Decateur, USA.

## Abstract

**Objective:**

To describe a crowdsourced disease surveillance project (EpiCore) and evaluate its usefulness in obtaining information regarding potential disease outbreaks.

**Methods:**

Volunteer human, animal and environmental health professionals from around the world were recruited to EpiCore and trained to provide early verification of health threat alerts in their geographical region via a secure, easy-to-use, online platform. Experts in the area of emerging infectious diseases sent requests for information on unverified health threats to these volunteers, who used local knowledge and expertise to respond to requests. Experts reviewed and summarized the responses and rapidly disseminated important information to the global health community through the existing event-based disease surveillance network, ProMED.

**Findings:**

From March 2016 to September 2017, 2068 EpiCore volunteers from 142 countries were trained in methods of informal disease surveillance and use of the EpiCore online platform. These volunteers provided 790 individual responses to 759 requests for information addressing unverified health threats in 112 countries; 361 (45%) responses were considered to be useful. Most responses were received within hours of the requests. The responses led to 194 ProMED posts, of which 99 (51%) supported verification of an outbreak, were published on ProMED and sent to over 87 000 subscribers.

**Conclusion:**

There is widespread willingness among health professionals around the world to voluntarily assist efforts to verify and provide supporting information on unconfirmed health threats in their region. By linking this member network of health experts through a secure online reporting platform, EpiCore enables faster global outbreak detection and reporting.

## Résumé

**Objectif:**

Décrire un projet participatif de surveillance des maladies (EpiCore) et évaluer son utilité pour obtenir des informations sur d'éventuelles flambées épidémiques.

**Méthodes:**

Des professionnels bénévoles de la santé humaine, animale et environnementale du monde entier ont été recrutés dans le cadre du système EpiCore. Ces professionnels ont été formés pour effectuer une vérification rapide des alertes sanitaires dans leur région géographique par l'intermédiaire d'une plate-forme en ligne sécurisée et facile à utiliser. Des experts des maladies infectieuses émergentes ont envoyé des demandes d'informations sur des menaces sanitaires non vérifiées à ces bénévoles, qui ont utilisé les connaissances et l'expertise disponibles localement pour y répondre. Les experts ont examiné et synthétisé les réponses fournies, puis ont rapidement diffusé les informations importantes à la communauté sanitaire mondiale par le biais de ProMED, un réseau de surveillance des maladies fondée sur des faits.

**Résultats:**

Entre mars 2016 et septembre 2017, 2068 bénévoles EpiCore originaires de 142 pays différents ont été formés aux méthodes de surveillance informelle des maladies et à l'utilisation de la plate-forme en ligne EpiCore. Ces bénévoles ont fourni 790 réponses individuelles aux 759 demandes d'informations portant sur des menaces sanitaires non vérifiées dans 112 pays; 361 (45%) réponses ont été jugées utiles. La plupart des réponses ont été reçues quelques heures après la demande. Les réponses ont donné lieu à 194 articles ProMED, dont 99 (51%) soutenaient la vérification d'une épidémie et ont été publiés sur ProMED et envoyés à plus de 87 000 abonnés.

**Conclusion:**

De nombreux professionnels de santé du monde entier sont prêts à soutenir bénévolement les efforts visant à vérifier les menaces sanitaires non confirmées dans leur région et à fournir des informations complémentaires à leur égard. En reliant les membres de ce réseau d'experts de la santé par l'intermédiaire d'une plate-forme en ligne sécurisée, EpiCore permet d'accélérer la détection des épidémies mondiales et l'établissement de rapports.

## Resumen

**Objetivo:**

Describir un proyecto de control de enfermedades con participación pública (EpiCore) y evaluar su utilidad para obtener información sobre potenciales brotes de enfermedad.

**Métodos:**

Se reunieron a profesionales de salud humana, animal y medioambiental de todo el mundo para el proyecto EpiCore y se capacitaron para proporcionar verificación temprana de alertas de amenazas para la salud en su región geográfica a través de una plataforma segura, fácil de usar y en línea. Los expertos en el área de enfermedades infecciosas emergentes enviaron solicitudes de información sobre amenazas para la salud no verificadas a estos voluntarios, quienes utilizaron el conocimiento local y la experiencia para responder a las solicitudes. Los expertos revisaron y resumieron las respuestas y rápidamente distribuyeron información importante a la comunidad mundial de salud a través de la red de control de enfermedades basada en eventos existentes, ProMED.

**Resultados:**

Desde marzo de 2016 hasta septiembre de 2017, 2068 voluntarios de EpiCore de 142 países se formaron en métodos de control de enfermedades casuales y el uso de la plataforma en línea de EpiCore. Estos voluntarios proporcionaron 790 respuestas individuales a 759 solicitudes de información sobre amenazas para la salud no verificadas en 112 países; de estas respuestas, 361 (45%) se consideraron útiles. La mayoría de las respuestas se recibieron en cuestión de horas a partir de la solicitud. Las respuestas condujeron a 194 artículos de ProMED, de los cuales 99 (51%) respaldaron la verificación de un brote, se publicaron en ProMED y se enviaron a más de 87 000 suscriptores.

**Conclusión:**

Existe una amplia disposición entre los profesionales de la salud de todo el mundo para ayudar voluntariamente con los esfuerzos de verificación y respaldo de la información sobre amenazas para la salud no confirmadas en sus regiones. Al conectar esta red de miembros expertos de la salud mediante una plataforma de información en línea segura, EpiCore facilita la detección y comunicación más rápida de brotes en el mundo.

## ملخص

**الغرض:**

وصف مشروع مراقبة المرض الجماعي (إيبكور) والوقوف على مدى أهميته في تجميع المعلومات المتعلقة بالحوادث المحتملة لانتشار الأمراض.

**الطريقة:**

انضم متطوعون من المتخصصين في صحة الإنسان والحيوان والبيئة من جميع أنحاء العالم في مشروع إيبكور وخضعوا للتدريب للكشف المبكر عن الإنذارات المتعلقة بالمخاطر التي تهدد الصحة في منطقتهم الجغرافية عبر منصحة آمنة سهلة الاستخدام عبر الإنترنت. ويرسل الخبراء في مجال الأمراض المعدية الناشئة طلبات للحصول على معلومات بشأن المخاطر الصحية غير المثبتة إلى المتطوعين، والذين استعانوا بدورهم بالخبرة والمعرفة المحلية للرد على هذه الطلبات. وقام الخبراء بمراجعة تلك الردود وتلخيصها ونشر المعلومات الهامة سريعًا في أوساط المجتمع الصحي العالمي عن طريق الشبكة الحالية لمراقبة الأمراض المعتمدة على الوقائع، والمعروفة باسم ProMED.

**النتائج:**

في الفترة من مارس/آذار 2016 حتى 10 سبتمبر/أيلول 2017، خضع 2068 متطوعًا تابعًا لإيبكور من 142 دولة للتدريب على طرق مراقبة الأمراض بشكلٍ غير رسمي واستخدام منصة إيبكور على الإنترنت. وقدم المتطوعون 790 ردًا فرديًا على 759 طلبًا للمعلومات فيما يتعلق بالمخاطر الصحية غير المثبتة في 112 دولة؛ وتم اعتبار 361 ردًا (بنسبة 45‏%) ضمن الردود المفيدة. وتم استلام أغلب الردود خلال ساعات من إرسال الطلبات. ونتج عن تلك الردود نشر 194 منشورًا تابعًا لـ ProMED، حيث قدم 99 (‏51%) منها دليلاً على تفشي المرض، وتم نشرها على ProMED وإرسالها إلى أكثر من 87 ألف مشترك.

**الاستنتاج:**

هناك استعداد واسع النطاق بين الأخصائيين الصحيين حول العالم لتقديم مساعدات في الجهود المبذولة طوعًا للكشف وتقديم معلومات تثبت وجود مخاطر صحية غير أكيدة في منطقتهم. وبفضل الاتصال بشبكة الأعضاء المكونة من خبراء الصحة خلال منصة إبلاغ آمنة على الإنترنت، تعمل إيبكور على تمكين الكشف السريع عن تفشي الأمراض عالميًا والإبلاغ عنها.

## 摘要

**目的:**

旨在描述众包疾病监测项目 (EpiCore) 并评估其在获取潜在疾病突发信息方面的有用性。

**方法:**

从世界各地招募人类、动物和环境卫生方面的专业人员志愿者至 EpiCore，并且通过安全、易于使用的在线平台让志愿者在其所处的地理区域接受培训，从而提供公共卫生威胁警报的早期证实。新兴传染病领域的专家向这些志愿者发出针对尚未证实卫生威胁的信息请求，这些志愿者得以运用其对当地情况的了解和专业知识，对相应请求做出回应。专家对这些回应进行审查和归纳，同时通过现有基于事件的疾病监测网络——ProMED，将重要信息迅速传播至全球卫生社区。

**结果:**

2016 年 3 月至 2017 年 9 月，来自 142 个国家的 2068 名 EpiCore 志愿者接受了非正式疾病监测以及 EpiCore 在线平台使用的培训。这些志愿者针对 759 份关于解决 112 个国家尚未证实卫生威胁的信息请求，提供了 790 份个人回答，其中 361 (45%) 份回答显示为有用。大多数回应能够在信息请求发出后数小时之内收到。这些回应生成了 194 份 ProMED 帖子，其中 99 (51%) 份为疾病突发的证实提供了支持。这些帖子被发布在 ProMED 上并被发给 87000 多名订阅者。

**结论:**

在全世界的卫生专业人员中，很多人愿意主动协助，提供其所在地区尚未确定卫生威胁的证实情况和支持信息。通过一个安全的在线报告平台连接卫生专家成员网络，EpiCore 加快了全球疾病突发的监测与报告。

## Резюме

**Цель:**

Описать проект для наблюдения и контроля заболеваний (EpiCore), организованный по принципу краудсорсинга, и оценить его практическую ценность в получении информации о потенциальных вспышках заболеваний.

**Методы:**

Компанией EpiCore со всего мира были на добровольных началах набраны специалисты по охране здоровья человека, животных и окружающей среды, которые прошли подготовку по обеспечению ранних проверок предупреждений об угрозах для здоровья в своем географическом регионе с помощью безопасной и простой в использовании онлайн-платформы. Эксперты в области возникающих инфекционных заболеваний направили запросы на получение информации о непроверенных угрозах здоровью этим добровольцам, которые использовали местные знания и опыт для ответа на запросы. Эксперты рассмотрели и обобщили ответы и быстро распространили важную информацию среди мирового сообщества специалистов здравоохранения с помощью существующей, основанной на событиях сети мониторинга заболеваний ProMED.

**Результаты:**

С марта 2016 года по сентябрь 2017 года 2068 добровольцев компании EpiCore из 142 стран прошли обучение методам неофициального наблюдения и контроля заболеваний и использованию онлайн-платформы EpiCore. Эти добровольцы предоставили 790 индивидуальных ответов на 759 запросов на получение информации, касающейся непроверенных угроз здоровью в 112 странах; 361 ответ (45%) был признан полезным. Большинство ответов было получено в течение нескольких часов после поступления запроса. Ответы послужили основой для публикации в ProMED 194 сообщений, отправленных более чем 87 000 подписчиков, в 99 из этих сообщений (51%) была предоставлена подтверждающая информация о вспышках заболеваний.

**Вывод:**

Специалисты в области здравоохранения во всем мире проявляют готовность добровольно содействовать усилиям по проверке и предоставлению вспомогательной информации о неподтвержденных угрозах для здоровья в их регионе. Используя эту сеть экспертов в области здравоохранения с помощью безопасной онлайн-платформы для сообщения данных, EpiCore обеспечивает более быстрое глобальное обнаружение вспышек заболеваний и составление отчетности.

## Introduction

Rapid detection of infectious disease events of potential public health concern allows for control measures to be implemented in a timely manner, thus limiting the size and geographical spread of outbreaks. Traditional disease surveillance is the continuous systematic collection, analysis and evaluation of health information from formal sources. These systems often rely on health-care professionals and diagnostic laboratory data to report information on the illnesses seen in clinical practice, in what is referred to as passive disease surveillance. This information is generally collected, analysed and disseminated by local health authorities, who report to centralized health ministries.^1^ While the information received is often highly reliable and verified within a uniform structure for reporting, traditional disease surveillance techniques face several challenges. These include: missing information from populations who do not access health care or do so through informal channels; unsuitability to detect new, potentially high-impact outbreaks (such as severe acute respiratory syndrome, avian influenza, Middle East respiratory syndrome); and delays in reporting due to difficulties in obtaining specimens for testing or due to delayed laboratory confirmation.

Informal sources or event-based surveillance systems complement formal public health mechanisms in the detection of outbreaks of infectious diseases in humans and animals.^2^^,^^3^ Innovations in disease surveillance can help fill some of the gaps in traditional surveillance by gathering health information from informal sources, such as social media, word of mouth and local news media, and sharing that information publicly and transparently. The Program for Monitoring Emerging Diseases (ProMED), a programme of the International Society for Infectious Diseases, pioneered innovative disease surveillance using the internet and has provided event-based surveillance for emerging infectious diseases since 1994.^4^^,^^5^ The inherent transparency and variety of sources available for event-based surveillance have been credited with speeding up the discovery and public communication of outbreaks.^6^ However, signals for event-based surveillance, which include media and first-hand reports from astute observers, are often initially unverified by public health authorities or laboratory results. Disease control measures may be delayed while waiting for confirmation.^7^ As an early warning system, ProMED frequently issues preliminary reports of potential public health events as a request for information in an attempt to elicit further verification of these events. However, these requests for information are sent to the entire ProMED membership (> 87 000 subscribers) and our internal data show that they go unanswered up to 95% of the time.

In 2013, four global public health organizations ‒ the International Society for Infectious Diseases, the Skoll Global Threats Fund, HealthMap and the Training Programs in Epidemiology and Public Health Interventions Network ‒ formed a partnership to set up the EpiCore global disease surveillance project. Aligning with the World Health Organization’s (WHO) interest in countries developing event-based surveillance systems, EpiCore utilizes crowdsourcing to gather supplementary information to validate rumours and informal reports about disease outbreaks. In this paper we describe the EpiCore disease surveillance project, and analyse data from the first 18 months of its implementation to evaluate its usefulness in obtaining information about potential disease outbreaks.

## Methods

### Concept

EpiCore draws on the local knowledge of a global community of volunteer human, animal and environmental health professionals (called responders) to verify reports from formal and informal sources on disease outbreaks in their own geographical region. They respond to requests for information sent by ProMED moderators (called EpiCore requesters) via a secure online networking and reporting system. The information collected through EpiCore is then shared with ProMED subscribers worldwide, many of whom work in government ministries of health and WHO.

### Implementation

Criteria were established for becoming a volunteer EpiCore responder. Responders had to have at least two of the following: an advanced degree in public health or a related field (such as master in public health); health professional certificate or licence (such as doctor of medicine, doctor of veterinary medicine, registered nurse); 3–5 years of experience in human or animal health; current affiliation with a medical centre, university, health ministry, department of health or other health-related organization including nongovernmental organizations (NGOs) and private sector organizations; or successful completion of a field epidemiology training programme. Responders were recruited at Training Programs in Epidemiology and Public Health Interventions Network (TEPHINET) global and regional meetings; at other infectious diseases meetings, such as the International Congress on Infectious Diseases (where more than 20% of attendees are in decision-making positions); through targeted recruitment of members of related organizations; and through online recruitment efforts using social media networks and the mailing lists of the partner organizations. The EpiCore programme manager vetted the applications from responders to ensure they met the above criteria. EpiCore responders were volunteers and obtained no financial compensation for their participation.

When EpiCore was implemented, ProMED was using the services of 47 consultants with expertise in the areas of infectious diseases, virology, public health or epidemiology, microbiology, veterinary medicine, plant biology and toxicology. Located around the world, these ProMED moderators were deployed to continuously collect and analyse formal and informal reports of potential outbreaks in humans, animals and agricultural plants. Formal information sources included reports and information from health ministries, WHO, academic institutions and NGOs. Informal sources could be articles in the local press or media, reports from individual clinicians or field-based NGO staff, as well as internet-based media such as discussion forums, blogs and social networking sites. When a requester discovered an unverified report of a potential outbreak, he or she used the EpiCore online platform to send a request for information to a selected group of EpiCore responders in a geographical region. Responders received the request in an email and used their on-the-ground knowledge and professional expertise to respond via the online platform. If the requester judged that the information was useful, he or she prepared a ProMED posting using the information provided by the responder and the level of confidentiality requested. This was posted to the ProMED website (http://www.promedmail.org) and sent in an email to ProMED’s more than 87 000 subscribers from 195 countries. EpiCore was listed as the source of information in ProMED reports unless the responder preferred to be more specifically identified. 

A digital platform was developed by HealthMap (Boston Children’s Hospital, Boston, United States of America) with input from the International Society for Infectious Diseases and other partners, to allow secure communication between requesters and responders. When making a request for information the requester was able to select EpiCore responders by country or by using a bounding box on the online platform that showed the approximate location of potential responders as pins on a map. To maintain confidentiality, responders were identified in the platform by a unique responder identification number. The requester was not able to identify the responders unless the responders chose to include their name and information when responding to a request for information. Responders had three choices about how their response would be used in a subsequent ProMED post: (i) paraphrase or do not use direct quotes from this response, mask any identifying information referenced in the response text and do not provide any details on my identity; (ii) quote this response, but mask identifying information referenced in the response text and do not provide any details on my identity; (iii) quote this response and attribute it to me. 

Secure shell protocol was used to authenticate and establish a secure connection between the participating EpiCore responder and project servers. Secure shell protocol is a commonly used encrypted network protocol to allow secure connections over unsecure networks such as the internet. The EpiCore data transferred to the EpiCore cloud server, which was then stored in the EpiCore database (MySQL) on an Amazon web services relational database service. The database was accessed only from the elastic compute cloud server over a transmission control protocol connection using MySQL, port 3306. The secure connection required a password, known only by project developers, to connect to the database.

Responders received training about innovative disease surveillance methods (the use of informal or unstructured data such as media reports and rumours), techniques for validation of information and the use of the online digital platform for responses. The training took about one hour and prospective EpiCore members could complete the course online or in-person at ISID conferences or TEPHINET workshops. Trainings were also held for requesters, both in-person and online, about how to generate requests for information, use the digital platform to receive responses, safeguard the confidentiality of responses and disseminate responses that either validated or de-escalated unverified outbreaks.

### Project evaluation

We used data from the EpiCore platform database since its inception through to September 2017 and analysed it using Excel (Microsoft Office 2011, Microsoft Corp., Redmond, USA). These data included information on both the EpiCore responders and the requests for information that had been initiated. Demographic information on EpiCore responders included geographical location, education level, professional background, years of experience and work sector. Data on the requests for information included the geographical location where the requests were sent, number of members reached, number of responses received, rating of usefulness of member responses and time from request for information initiation to first response. Requesters graded responses as useful if they contributed information supporting, providing additional data or refuting an event. Each response that led to a ProMED report was evaluated by two of the authors to determine whether a report supported verification (indicated laboratory or other confirmatory data supporting the diagnosis), refuted verification (negative laboratory data or finding of an alternative diagnosis) or provided additional information (such as serotype or information on extent of an outbreak). Some EpiCore reports provided no new information and were categorized as such. Duplicate reports, such as those from ProMED regional networks, were combined and considered as a single report.

## Results

Between March 2016 and September 2017, 2068 human, animal and environmental health professionals from 142 countries applied and trained in informal disease surveillance and the use of the EpiCore platform. The majority of EpiCore responders self-identified as having a professional background in human health (1039, 54%), followed by human and environmental health (329, 17%), animal health (215, 11%), human and animal health (142, 7%), human and animal and environmental health (127, 7%), environmental health (40, 2%) and animal and environmental health (35, 2%). About half of current EpiCore responders (1073, 52%) had more than 10 years of experience in their respective field of work. Another 530 (26%) had 5–10 years of experience, 298 (14%) had 3–5 years of experience and 167 (8%) had less than 3 years of experience. Most EpiCore responders (1259, 61%) worked in the government sector, 488 (24%) in NGOs or the non-profit sector and 320 (15%) in the private sector.

**Fig. 1:**
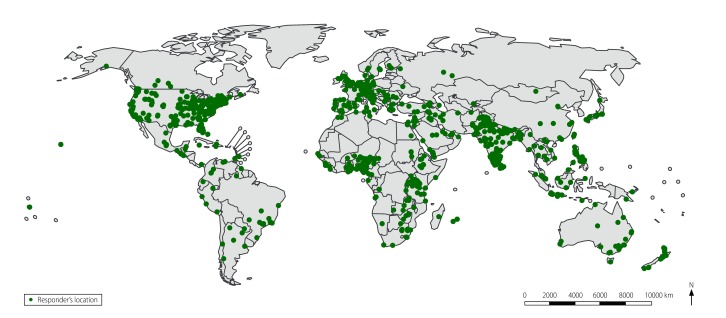
Locations of 2068 responders in 142 countries in the EpiCore disease surveillance project, November 2015 to September 2017

From March 2016 to September 2017, requesters sent 759 requests for information concerning unverified outbreak alerts to EpiCore responders. Requests for information addressed events in over 100 countries (Fig. 1), had 15 120 views by a member and elicited 1923 responder responses. Of the requests for information, 466 (61%) received at least one response (Table 1). The time from issuing the request to receiving the first response ranged from 1 minute to 62 days with a median of 6 hours. Of the 790 individual responses, 361 (45%) were considered to be useful by the requester and led to a ProMED post to subscribers. Examples of useful responses to requests for information received via EpiCore are shown in Box 1. EpiCore responses informed 194 ProMED posts to subscribers: 99 (51%) supported verification, 56 (29%) provided additional information, 23 (12%) refuted verification and 16 (8%) provided no new information.

Data Source: EpiCore Disease Surveillance Project.

**Table 1:** Distribution by country of responders, requests for information and responses in the EpiCore disease surveillance project, November 2015 to September 2017

Country	No. of requests for information sent	Total no. of EpiCore responders in country	No. of responses to requests for information	No. (%) of requests for information with at least one response	No. (%) of useful^a^ responses
India	121	186	91	60 (50)	72 (79)
United States	78	399	38	51 (65)	37 (97)
Pakistan	51	86	97	37 (73)	70 (72)
Iraq	43	20	29	22 (51)	24 (83)
Egypt	30	18	18	17 (57)	17 (94)
Saudi Arabia	30	12	9	13 (43)	6 (67)
Syrian Arab Republic	30	0^b^	0	19 (63)	0 (0)
Nigeria	19	189	176	19 (100)	171 (97)
Bangladesh	16	33	11	8 (50)	10 (91)
Brazil	14	32	21	11 (79)	11 (52)
Angola	12	3	0	0 (0)	0 (0)
Nepal	12	25	21	6 (50)	17 (81)
Israel	11	7	9	7 (64)	7 (78)
South Africa	10	19	8	7 (70)	8 (100)
United Kingdom	10	61	15	9 (90)	11 (73)
Russian Federation	9	7	4	5 (56)	3 (75)
Kazakhstan	8	1	0	2 (25)	0 (0)
Kenya	8	25	8	6 (75)	5 (63)
Myanmar	8	4	3	2 (25)	3 (100)
Philippines	8	77	10	8 (100)	10 (100)
All other countries	231	719	790	157 (68)	187 (93)
**Total**	**759**	**1923^c^**	**1358**	**466 (61)**	**669 (87)**

^a^ Useful responses are identified by the requester when a response contributes information supporting, providing additional data or refuting an event.

^b^ Requests for information may be sent to respondents in neighbouring countries if there are no members in the country where the event is occurring.

^c^ The total number of EpiCore responders in this table includes only those members in countries where a request for information was sent. This table lists the 20 countries with the most requests for information received.

Box 1 **Examples of useful responses to requests for information received by the EpiCore disease surveillance project, November 2015 to September 2017**

- A report on a polio case in Gilgit-Baltistan, Pakistan, was received by ProMED, which was the first case reported from that area since 2011. A request for information was sent to EpiCore responders to identify whether the poliovirus involved in this case was a wild poliovirus or vaccine derived. Within one hour, a responder confirmed the case was due to a wild poliovirus with genetic linkages to a virus identified in environmental samples in Lahore from the previous year.^8^^,^^9^
- ProMED received a report from a media source about a localized potential infectious disease outbreak and a request for information was sent to over 100 nearby EpiCore responders. The request for information received eight responses that not only confirmed the outbreak, but also alerted a local government official who had yet to hear about the outbreak and reported that a team had been sent to investigate.^10^ (The country and disease information has been omitted to maintain requested confidentiality of the EpiCore responders).
- After receiving a report from a media source about cholera in Zambia, ProMED sent a request for information to EpiCore responders asking for more information and updated numbers. Within 10 minutes, an EpiCore responder confirmed 11 cases and the following day this number was confirmed by another EpiCore responder.^11^
- ProMED received a report of suspected Crimean–Congo haemorrhagic fever in Pakistan. A request for information was sent to EpiCore responders in Pakistan and within less than an hour a responder replied refuting the diagnosis.^12^

ProMED: Program for Monitoring Emerging Diseases of the International Society for Infectious Diseases.

## Discussion

Innovative disease surveillance methods are already being used to speed up the detection of public health threats, some of which have global importance. ProMED, HealthMap and other systems that rapidly detect outbreaks digitally through unofficial information sources are widely used by those in charge of responding to outbreaks.^13^ However, unofficial data may consist of unverified information (sometimes referred to as rumours), and actions to control outbreaks may be delayed pending further verification. In many cases, initial reports of potential public health events are found in media reports that do not mention confirmation from government sources. EpiCore complements current ministry of health and WHO systems by addressing these gaps and verifying or refuting numerous unconfirmed public health events. In many other cases, EpiCore responders were able to provide supplementary data that could assist in formulating outbreak response efforts.

Analysis of nearly 200 ProMED reports during the study period citing EpiCore data indicated that, in most cases, the report was validated. In other cases, alternative explanations for events or negative test results were found or additional data concerning the event were provided and posted to ProMED subscribers. The purpose of epidemic intelligence is to allow timelier public health action towards containment, disease control and other measures that may reduce the size and geographical spread of an outbreak. EpiCore has shown the ability to improve the verification (or refutation) of unconfirmed outbreaks and to identify the etiology of unknown outbreaks. While such additional information may not provide absolute verification, we believe that at times it will be sufficient to allow public actions to mitigate potentially dangerous situations.

The finding that 759 requests for information were generated (more than one per day during the study period), and that 61% of these received at least one response deemed useful by the requester, indicates the potential value of EpiCore in validating outbreak reports. More than half of the responses validated the request for information, supporting the usefulness of event-based surveillance. While we were not able to determine the actions taken as a result of these reports, we hope to be able to collect this information in the future. However, actions undertaken as a result of such reports could reduce the size, geographical extent, morbidity, mortality and economic costs of outbreaks. Additional information obtained through EpiCore reports (such as serotypes or antimicrobial resistance of disease-causing organisms) could improve the quality of outbreak responses. Refutation of reports could reduce unnecessary actions and thus reduce costs to public health agencies.

Current challenges and limitations of EpiCore include the uneven geographical distribution of responders, with some countries and regions over-represented and others, including some hotspots for emerging diseases, under-represented. Some countries have no or very few responders. This is due in part to recruitment efforts being driven by the location of infectious disease conferences, ProMED subscribers and partner organizations. Not all requests for information received helpful responses and some requests for information generated more responses than could be easily used. Responses to requests for information are evaluated on their usefulness by the requester, which is subjective. A high level of expertise is required by the requester to properly frame the queries included in requests for information and to interpret responses meaningfully. Some responders, despite the promise of anonymity, expressed misgivings about providing responses when this conflicted with the confidentiality requirements of their other professional duties. Neither ProMED nor EpiCore respond directly to public health outbreaks and emergencies. The goal is to provide actionable information, but it is too early in the project to identify specific actions taken as a result of EpiCore responses. In the future, we will tabulate public health responses to events, although any action taken cannot necessarily be attributed to EpiCore since public health authorities could be acting on information received elsewhere.

During the project period, EpiCore was able to demonstrate the value of crowdsourcing for enhancing disease surveillance activities by showing the feasibility of recruiting and training highly qualified volunteer professionals around the world for outbreak validation and verification. A secure digital platform was developed that facilitated swift interaction between information requesters and EpiCore responders in targeted geographical areas, while safeguarding the confidentiality of EpiCore responders. Requesters could then rapidly and transparently disseminate additional information supporting or refuting the veracity of outbreaks through ProMED so that appropriate public health action could occur.

EpiCore does not aim to replace any official reporting system and cannot be considered verification in WHO terms as that would require confirmation by a member state’s public health authority, usually based on laboratory evidence. EpiCore is meant to be a complementary tool supporting traditional and official surveillance systems through crowdsourcing. As ProMED subscribers include many personnel from the WHO, World Organisation for Animal Health, national ministries of health and agriculture and many regional and local health authorities, the information EpiCore obtains reaches those subscribers. In many cases, the initial reports of events of potential interest to the health sector (both human and animal) are found in media reports that do not include confirmation from government sources. In those cases, the reports generated by responses may assist WHO and national governments in their own verification processes.

EpiCore software version 2.0 is now being developed and will attempt to address some of the limitations of the current system. The software will include enhancements to the user experience for both requesters and responders that we expect will lead to improved response rates and quality. We plan to provide greater engagement with responders through more frequent communication, additional requester groups and non-monetary incentives. Finally, we plan to recruit responders from regions not currently adequately represented and to include subject-matter expert responders who can help provide information on potential outbreaks regardless of geographical location. Results from this initial period will provide baseline rates against which future responses can be assessed. Ultimately, we believe this will contribute to faster outbreak detection and quicker responses, limiting the extent of outbreaks and preventing pandemics.^14^
